# Automatic monitoring and detection of tail-biting behavior in groups of pigs using video-based deep learning methods

**DOI:** 10.3389/fvets.2022.1099347

**Published:** 2023-01-11

**Authors:** Franziska Hakansson, Dan Børge Jensen

**Affiliations:** Department for Veterinary and Animal Science, Faculty of Health and Medical Science, University of Copenhagen, Copenhagen, Denmark

**Keywords:** convolutional neural network (CNN), long short-term memory (LSTM), tail-biting behavior, pigs, computer vision, animal welfare

## Abstract

Automated monitoring of pigs for timely detection of changes in behavior and the onset of tail biting might enable farmers to take immediate management actions, and thus decrease health and welfare issues on-farm. Our goal was to develop computer vision-based methods to detect tail biting in pigs using a convolutional neural network (CNN) to extract spatial information, combined with secondary networks accounting for temporal information. Two secondary frameworks were utilized, being a long short-term memory (LSTM) network applied to sequences of image features (CNN-LSTM), and a CNN applied to image representations of sequences (CNN-CNN). To achieve our goal, this study aimed to answer the following questions: (a) Can the methods detect tail biting from video recordings of entire pens? (b) Can we utilize principal component analyses (PCA) to reduce the dimensionality of the feature vector and only use relevant principal components (PC)? (c) Is there potential to increase performance in optimizing the threshold for class separation of the predicted probabilities of the outcome? (d) What is the performance of the methods with respect to each other? The study utilized one-hour video recordings of 10 pens with pigs prior to weaning, containing a total of 208 tail-biting events of varying lengths. The pre-trained VGG-16 was used to extract spatial features from the data, which were subsequently pre-processed and divided into train/test sets before input to the LSTM/CNN. The performance of the methods regarding data pre-processing and model building was systematically compared using cross-validation. Final models were run with optimal settings and evaluated on an independent test-set. The proposed methods detected tail biting with a major-mean accuracy (MMA) of 71.3 and 64.7% for the CNN-LSTM and the CNN-CNN network, respectively. Applying PCA and using a limited number of PCs significantly increased the performance of both methods, while optimizing the threshold for class separation did result in a consistent but not significant increase of the performance. Both methods can detect tail biting from video data, but the CNN-LSTM was superior in generalizing when evaluated on new data, i.e., data not used for training the models, compared to the CNN-CNN method.

## 1. Introduction

Most commercial pigs in the EU are raised under intensive conditions that are likely to increase the development of abnormal social behavior such as tail biting in groups of pigs (1). Tail-biting behavior, characterized by one pig orally manipulating another pig's tail, often develops in situations where behavioral needs are not met (2), and is considered an indicator of negative welfare of the animal receiving the behavior, as well as the animal performing it. The inflicted tail damage can range from superficial bites and minor scratches to severe wounds, which in severe cases can get infected. Secondary infections can result in partial or full loss of an individual's tail (3, 4) and carcass condemnation at slaughter (5). Pigs that are subjected to tail biting experience pain and stress (6), and may require medical treatment and space allotment in hospital pens. Tail-biting behavior is therefore considered both an economic and a welfare challenge in modern pig farming (7, 8).

Tail biting is a complex problem of multifactorial origin, and a wide range of risk factors linked to the behavior have been discussed, such as high stocking density, poor climatic conditions, inadequate feed and inadequate enrichment material [e.g., (3, 9–11)]. Current preventive strategies include the removal of risk factors that might trigger the development of biting behavior in groups of pigs, tail docking (i.e., surgically removing a part of the tail to hamper tail-biting behavior), supplying enrichment material, or a combination of those. However, routine tail docking is associated with procedural (12) and chronic pain (13) and is prohibited by European law (14). Additionally, intervention strategies are often considered unfeasible, costly, or time-consuming for the farmer. Hence, timely detection of the behavior and early intervention still seem to be the most feasible ways of preventing severe biting and potential outbreaks. However, as monitoring of tail biting is often done by direct observation, and intervention is often applied after the first tail wounds emerged or an outbreak has occurred, the onset of the behavior and smaller lesions are often overlooked. Moreover, direct observations are limited to specific times per day and are time-consuming and subjective. Hence, there seems to be a need for both continuous and automatic surveillance tools monitoring the development of tail-biting behaviors in groups of pigs. Such tools have the potential to aid pig farmers in their decision making on prevention and intervention strategies, thus reducing labor costs, and increasing overall animal welfare.

Previous studies have shown that tail-biting behavior and other oral and nasal manipulation directed toward pen-mates are connected (15–17), and that low level biting behavior can predict outbreaks of tail biting (18). Moreover, early tail biting was shown to be associated with increased activity, exploration behavior and a change in tail posture (17, 19–21), with the latter being proposed as an early detection method for tail-biting outbreaks (22–24). Using a precision livestock farming (PLF) approach, D'Eath et al. (25) investigated the automatic assessment of tail position in pigs with intact tails using 3D cameras mounted above the feeding area. In their study, the authors analyzed group level data on pigs' tail position using (proprietary) machine vision algorithms. Although the algorithm identified low hanging vs. not low hanging tails with 73.9% accuracy, the proposed algorithm was not able to detect a high tail positions or a curly tail, despite them being the majority tail postures. Moreover, although the authors reported an increase in low hanging tails prior to a tail-biting outbreak, tails were also found to be hanging low in situations not related to tail biting (e.g., moving of animals), indicating that tail position might not be the most reliable indicator of tail biting. Larsen et al. (26) attempted to predict tail-biting events using sensor data on water usage and temperature at both ends of the pen, using dynamic linear models and artificial neural networks. The authors developed several models using the different data sources, both independently and in combination, and found that when tested in a real-life setting a combination of data on water usage and pen temperature resulted in the highest performance [area under the curve (AUC) = 0.769]. However, both the sensitivity and the specificity of the model were only moderate, due to a high level of false alarms being caused by the pigs' drinking behavior changing in response to problems other than tail biting.

Within the last decade, several studies have reported on the use of video-based automatic monitoring systems to detect and classify specific behaviors in pigs, such as aggressive behavior (27–29), nursing behavior (30), feeding/drinking behavior (31), enrichment use (32) and tail-biting behavior (33). While earlier studies on image analyses implemented e.g., Linear Discriminant Analysis on motion history images (28) or used data on the activity index between subsequent frames as input into a multilayer feed forward neural network (29), the majority of the above studies applied deep machine learning algorithms. However, simple feed-forward neural networks utilizing single frame image data is not sufficient to capture the temporal component of fast occurring and rare behaviors, such as tail-biting behavior (34). To account for temporal information in behavioral data, recurrent neural networks (RNN) able to analyse sequential data are implemented [e.g. (32, 33)], often in combination with tracking algorithms. In their study, Liu et al. (33) investigated tail-biting behavior in group-housed pigs using tracking of pairwise interactions and subsequently analyzing the image data using a convolutional neural network (CNN) combined with a long short-term memory (LSTM) network. Although the authors were able to develop a model that identified 89.23% of the tail-biting interactions correctly, the performance of a classification model is heavily dependent on the output of a tracking model. Moreover, combining varying modeling approaches increases the computational workload needed to detect biting behavior, which could make an on-farm implementation more difficult. It seems reasonable to assume that simplifying algorithms and optimizing these to the problem at hand is valuable to the farmer.

Another method for reducing the computational workload is by dimensionality reduction of the data being used in the models. Principal component analysis (PCA) is a method used for dimensionality reduction in many different situations [e.g., (35, 36)]. When PCA is applied, data with a given set of dimensions (axis) are transformed to new coordinate system with an identical set of new axes, where the order of the axis corresponds to the amount of information that is contained in that axis alone. Dimensionality reduction is thus achieved by only using the first *N*_*PC*_ dimensions of the PCA-transformed data, which contain sufficient information for a given purpose.

The current study aims at developing a video-based deep learning approach for detecting tail-biting behavior in groups of pigs without the implementation of a prior tracking algorithm. To achieve this aim, we intend to apply a pre-trained CNN for extraction of latent features from the video frames, combined with two secondary models to analyse sequential data. Specifically, the two secondary models are a LSTM network applied to sequences of the extracted image features (CNN-LSTM) and a CNN applied to image representations of extracted spatial features (CNN-CNN). Further, the optimal strategy regarding data pre-processing and model architecture resulting in the best performance for the given data will be determined for the two models.

## 2. Material and methods

### 2.1. Brief overview

**Figure 1:**
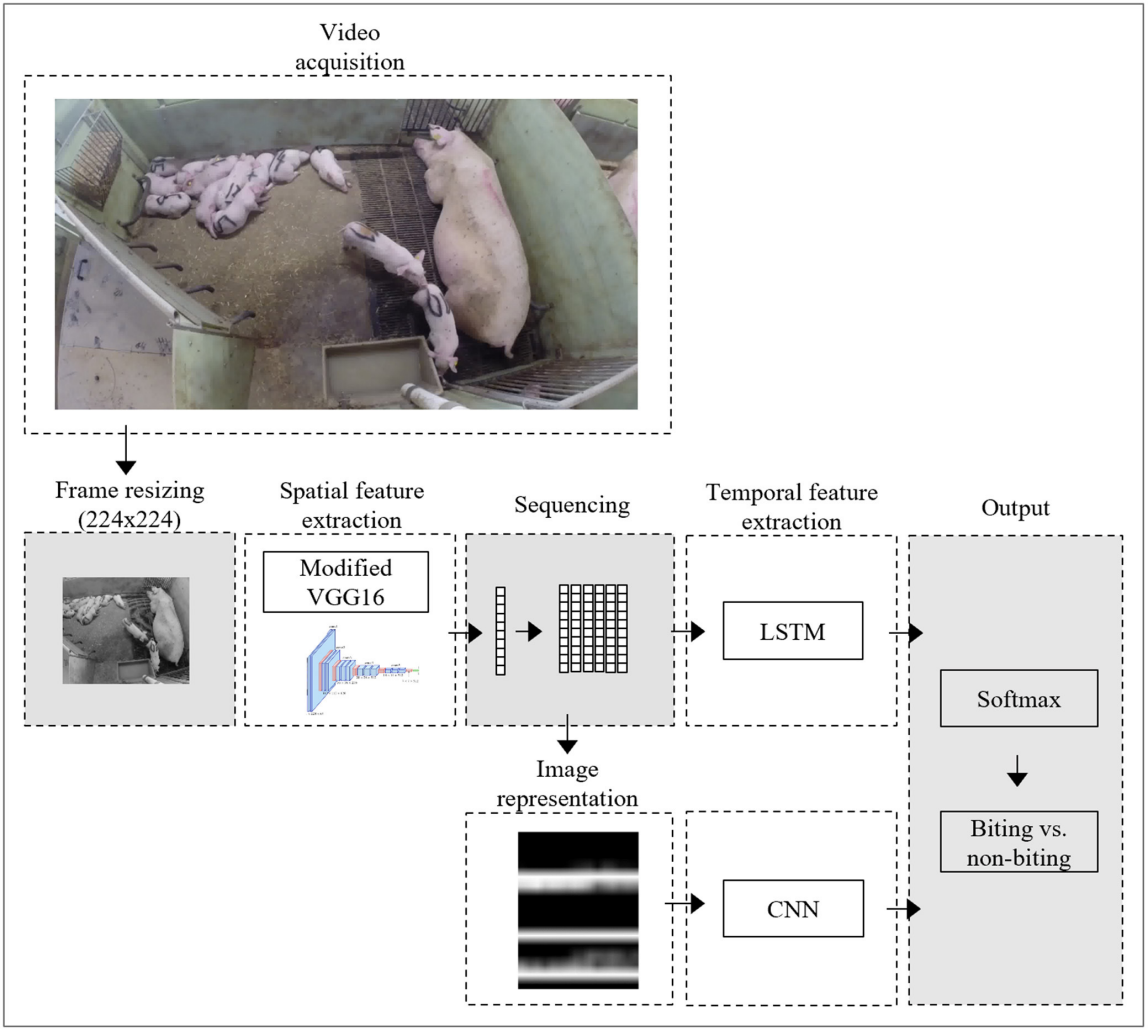
Schematic illustration of the processing approach, showing the steps from video acquisition to output generation for the CNN-LSTM and CNN-CNN networks.

This subsection gives a brief overview of the approach taken in this study (Figure 1). The subsequent subsections will elaborate with full details.

1. A pre-trained CNN was used to transform video images into a 4096-dimensional latent feature vector.
2. Feature vectors of sequential images were combined into sequences and input into two secondary neural networks, which would classify each sequence as either containing or not containing a biting event. Two different types of secondary networks were used, namely LSTM and CNN. Different hyper-parameter settings were assessed using 4-fold cross-validation to determine the set of parameters resulting in the highest performance, based on data from eight of the 10 pens included in this study.
3. Using the best set of parameters, final LSTM and CNN network were trained based on the entire dataset and tested on data from two pens, which had been held out from previous trainings.

### 2.2. Experimental set-up and video acquisition

The video data used in this study was collected at a commercial Danish piggery under the approval of the farm owner/manager during a previous study (21). The data were collected without interfering with the general on-farm management. Ethical review and approval was not required for the study on animals in accordance with the local legislation and institutional requirements.

The pigs [(Landrace × Yorkshire) × Duroc] used in this study were piglets prior to weaning (30 ± 1.6 days of age) and were housed with their dams in free-farrowing pens. All details on housing and management can be found in a previous publication (16). Video data were acquired from 26 pens, although only a subsample of 10 pens were used for the purpose of this study. The included videos were selected based on the following inclusion criteria: (1) no interference with the stockperson working in the pen, (2) minimum recording of 55 min and (3) piglets were visible for more than 80 % of the video recording.

A GoPro Hero 3+ camera (Silver edition, hard-box case, GoPro^®^ Inc.) was fixed approximately 2 m above the floor and recorded the entire pen area. Video recordings were limited to the open area of the pen, and piglets in the creep were not visible. The camera recorded RGB videos with a resolution of 1,920 × 1,080 pixels in MPEG4 format and a frame rate of 59.94 frames per second (fps). Videos were recorded prior to the weaning of the piglets and each pen was recorded once for 60 min. Recordings took place either in the morning (video start: 09:00) or the early afternoon (video start 12:00) at randomized order to minimize disturbances by farm staff. All video data were manually labeled by a single observer using all occurrence sampling. Biting behavior was labeled according to the ethogram of Hakansson and Bolhuis (21) and was assessed individually for all piglets within a pen (see Table 1). Both tail biting and tail-in-mouth behavior were assessed to include milder as well as more severe incidences of tail biting. For later analysis, the two biting behaviors have been combined.

**Table 1:** Ethogram of biting behavior in piglets, adapted from Hakansson and Bolhuis (21).

**Behavior**	**Description**
Tail biting	Nibbling, suckling, or chewing at the tail of a pen mate, causing a reaction from the other pig.
Tail-in-mouth	Gentle nibbling, suckling, or chewing of another pig's tail, without causing a reaction in the other pig.

The computer used to run all codes was equipped with an Intel^®^ Xeon^®^ W-2235 CPU @ 3.80 GHz processor with 128 GB RAM and an NVIDIA RTX R4000 16 GB graphics card. The operating system running on the machine was Microsoft Windows Enterprise 10. The software used to implement the algorithms was R Version 3.6.1 (37) and the library KERAS (Version 2.9.0).

### 2.3. Data description and pre-processing

A total of 208 unique biting events were present in the dataset. Descriptive analysis of the data in this study revealed that tail biting durations ranged from 1 to 45s, with 55 % of the tail-biting events lasting between 1 to 4s. From the continuous video data, still frames were extracted with a framerate of 10 fps, and the images were subsequently connected with their respective metadata. The data were manually post-processed and frames with obstructions (e.g., due to dirt) were removed. The full dataset consisted of 332.666 images, of which 5.330 images showed biting behavior (see Table 2).

**Table 2:** Descriptive statistics of dataset (CV, Cross-validation).

**Pen-ID**	**No. images without biting event**	**No. images with biting event**	**Unique tail-biting events**	**Dataset**	**CV test set**
1	29,436	900	32	Test	-
2	31,660	1340	52	Test	-
3	32,810	190	29	CV/Train	CV1
4	32,580	420	16	CV/Train	CV1
5	32,550	450	19	CV/Train	CV2
6	32,500	500	14	CV/Train	CV2
7	32,450	550	32	CV/Train	CV3
8	32,950	50	9	CV/Train	CV3
9	32,320	680	9	CV/Train	CV4
10	32,750	250	21	CV/Train	CV4
Total	327,336	5,330	208		

The datasets from each pen were divided into sub-sequences of varying lengths (10, 20, and 30 frames). Each sub-sequence was labeled based on whether or not it contained events of pigs performing tail-biting behavior. Sequences not showing incidences of tail biting did include other behaviors of pigs being in proximity or touching each other (e.g., negative social behavior such as play/fight, aggression and mounting, or nursing behavior).

The images were resized to the dimensions 224 × 224 × 1. For each resized image in the dataset, numeric latent feature representations were extracted using the pre-trained vision model VGG-16 (38), which was truncated after the first fully-connected layer. The truncated model outputted a 4,096-dimensional (1 × 1 × 4096) feature vector. Those latent features, which had a variance of 0 throughout the entire data set, were removed.

Subsequently, PCA was applied to the remaining latent features from all images from all pens simultaneously. In this study, we wanted to know how much the dimensionality of the latent feature data could be reduced via PCA before being used as inputs in the secondary models, without reducing the performance of these secondary models, compared to when all features are included. Thus, two data sets were used in the subsequent steps: the data set consisting of the latent feature vectors (without 0-variance) and the data set consisting of the principal components of these same feature vectors. Finally, min-max scaling was applied per feature in the data consisting of extracted latent features, and per principal component in the data consisting of the output from the PCA, to normalize the data to a range from 0 to 1.

### 2.4. Training of the secondary models

Data from two randomly selected pens were labeled as the outer test set and reserved for the assessment of the performance of the final models. Data from the remaining eight pens were used as the training data in a 4-fold cross-validation framework, where the data were iteratively split into inner training and test sets; in each iteration of the cross-validation, the inner training set would consist of all data from six of the pens, while the inner test set would consist of all data from the remaining two pens. Furthermore, data from one of the six pens in each inner training set was randomly selected to be removed from the inner training set and was instead used as the validation set. In this way, all models were trained, validated, and tested on mutually independent datasets. Early stopping was implemented during model training i.e., the training was stopped when the loss on the validation set had failed to decrease for 10 consecutive epochs. Before training, the inner training set was balanced using random under-sampling, so that the number of training sequences without tail biting would match the number of training sequences with tail biting. The inner validation ad test sets were not balanced.

### 2.5. Parameter tuning

Table 3 provides a brief overview of the parameters which were optimized using the learning set, and which values each parameter could take. The following two subsections elaborated on these parameters.

**Table 3:** Parameter specifications, which were compared by the cross-validation.

	**Name**	**Definition**	**Values**
Input dimensions	N_Obs_	Number of frames in each sequence	10, 20, 30
	N_PC_	Selected number of extracted principal components	4, 8, 16, 32, 64, 128, 256, 512, 3485
Model architecture	Layer	Number of LSTM/CNN layer in the secondary NN	1, 2, 3
	Nodes	Number of nodes divided over the LSTM layer	Mean (N_PC_, 2), mean (N_PC_, 2)/2, sum (N_PC_, 2)
	1^st^ Filter-size	Filter size of the first CNN layer	8, 16, 32, 64
	Dropout	Dropout rate applied during model training for CNN/LSTM	0, 0.1, 0.2

#### 2.5.1. Input dimensions

Different dimensions were tested and compared for the data used as inputs in the secondary models.

The first *N*_*PC*_ = 2^n^ [where n ∈ (2, 3, 4, 5, 6, 7, 8, 9)] principal components from the PCA-transformed latent features were extracted from each sequence, so that the dimensions of the inputs to the secondary models had the dimensions *N*_Obs_ × *N*_PC_ [where *N*_Obs_ ∈ (10, 20, 30)] for the PCA-transformed features. The untransformed feature data always had the dimensions *N*_Obs_ × *N*, where *N* is the number of latent features with a non-zero variance i.e., 3485. These data sequences were used directly as inputs for the LSTM and were converted to an image to be used as inputs for the secondary CNN.

#### 2.5.2. Model architectures

For both secondary models, different architectures where tested and compared.

For the LSTM model, LSTM layers were always followed by batch normalization. Similarly, for the CNN model each convolutional layer was always followed by batch normalization. The number of [LSTM + batch normalization] and [convolution + batch normalization] elements varied between 1, 2, and 3. For the LSTM model, the number of nodes in the LSTM layer was based on three different functions of the number of principle components used as the input, namely mean (N_PC_, 2), mean (N_PC_, 2)/2, and sum (N_PC_, 2), where 2 is the number of outputs in the secondary model.

**Figure 2:**
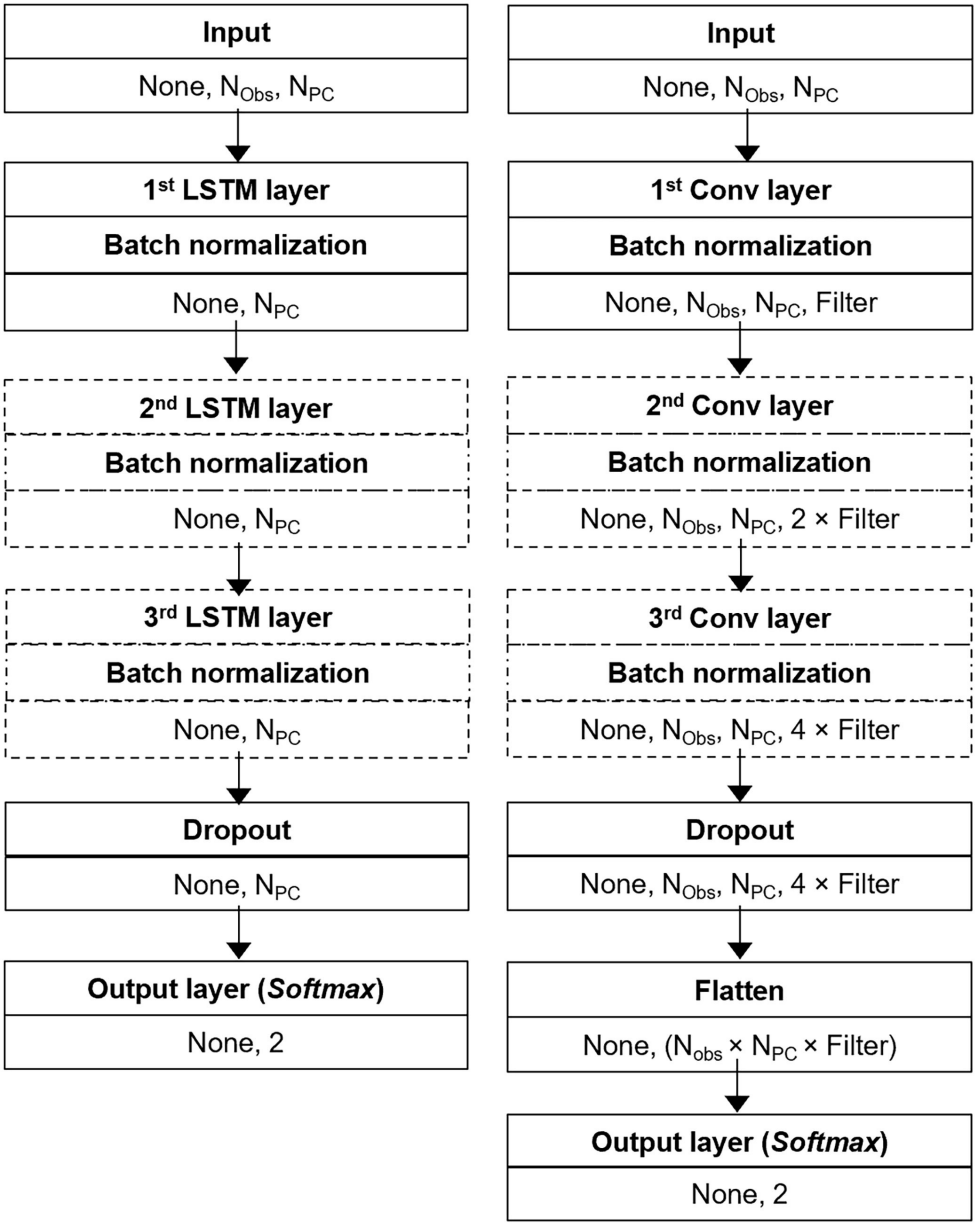
Schematic illustration of the general network architectures for the LSTM **(left)** and CNN **(right)** used as secondary models in this study. The dashed lines indicate elements which were only included in some variations of the architectures being tested and compared.

For the CNN model, the number of convolutional kernels (Filters) would double from the first to the second convolution, and double again from the second to the third convolution. The number of filters in the first convolution was varied, with possible values being 8, 16, 32, and 64. In all cases, the convolutional kernels had the dimensions 3 × 3. For the LSTM model, dropout was applied after the last [LSTM + batch normalization] element. Similarly for the CNN model, dropout was applied after the last [convolution + batch normalization] element. Both secondary models were trained with a batch size of 16. The optimization function *Adam* was used with an initial learning rate of 0.001. Figure 2 summarizes the architectures for the two secondary models.

### 2.6. Performance evaluation

For evaluating the performance of the trained models when applied to the inner and outer test sets, the major mean accuracy (MMA) was used in this study.

A positive prediction is seen when the positive probability for biting behavior, as outputted by the trained model, is above a set threshold. The value of the MMA will depend on the value of this threshold. Conventionally, this threshold is set to 0.5, which we in this study refer to as the naïve MMA (nMMA). In this study, however, we also wanted to investigate the potential for improving the performance by using different thresholds. To this end, we varied the threshold value between 0 and 1 by steps of 0.01. For each threshold value, the number of true positive predictions (TP), the number of false negative predictions (FN), the number of true negative predictions (TN), and the number of true positive predictions (TP) was calculated. These were then used to calculate the sensitivity, specificity, false positive rate (FPR), and MMA given the threshold, according to Equations 1–4 respectively.


(1) $$\begin{array}{l} Sensitivity\;=\;\frac{TP}{TP\;+\;FN} \end{array}$$

(2) $$\begin{array}{l} Specificity\;=\;\frac{TN}{TN\;+\;FP} \end{array}$$

(3) $$\begin{array}{l} FPR\;=\;1\;-\;Specificity \end{array}$$

(4) $$\begin{array}{l} MMA\;=\;mean\;\left(Sensitivity,\;Specificity\right) \end{array}$$

The threshold which resulted in the best MMA was identified, and the corresponding best MMA (btMMA) was saved.

The set of parameter settings, which resulted in the highest nMMA and btMMA, respectively, were identified in each iteration of the cross-validation. Furthermore, the effects of each possible value of each parameter setting on nMMA and btMMA were estimated using linear mixed-effect models (LMEM) with nMMA and btMMA, respectively, as the dependent variable. Each LMEM included pen-ID as a random effect, and the varying parameter of the data pre-processing and model architecture as the independent variables.

By combining the information from best set of parameters and the estimated effect of different values of all parameters, a final set of parameters were selected. Using this final set of parameters, the final versions of the secondary LSTM and the CNN model was trained on the entire outer training set and was tested on the outer test set. The nMMA and btMMA was identified for the outer test set. In addition, receiver operating characteristics (ROC) curves were made by plotting the Sensitivity against the FPR, and the area under this curve (AUC) was calculated as an additional performance metric for the outer test set.

## 3. Results

**Figure 3:**
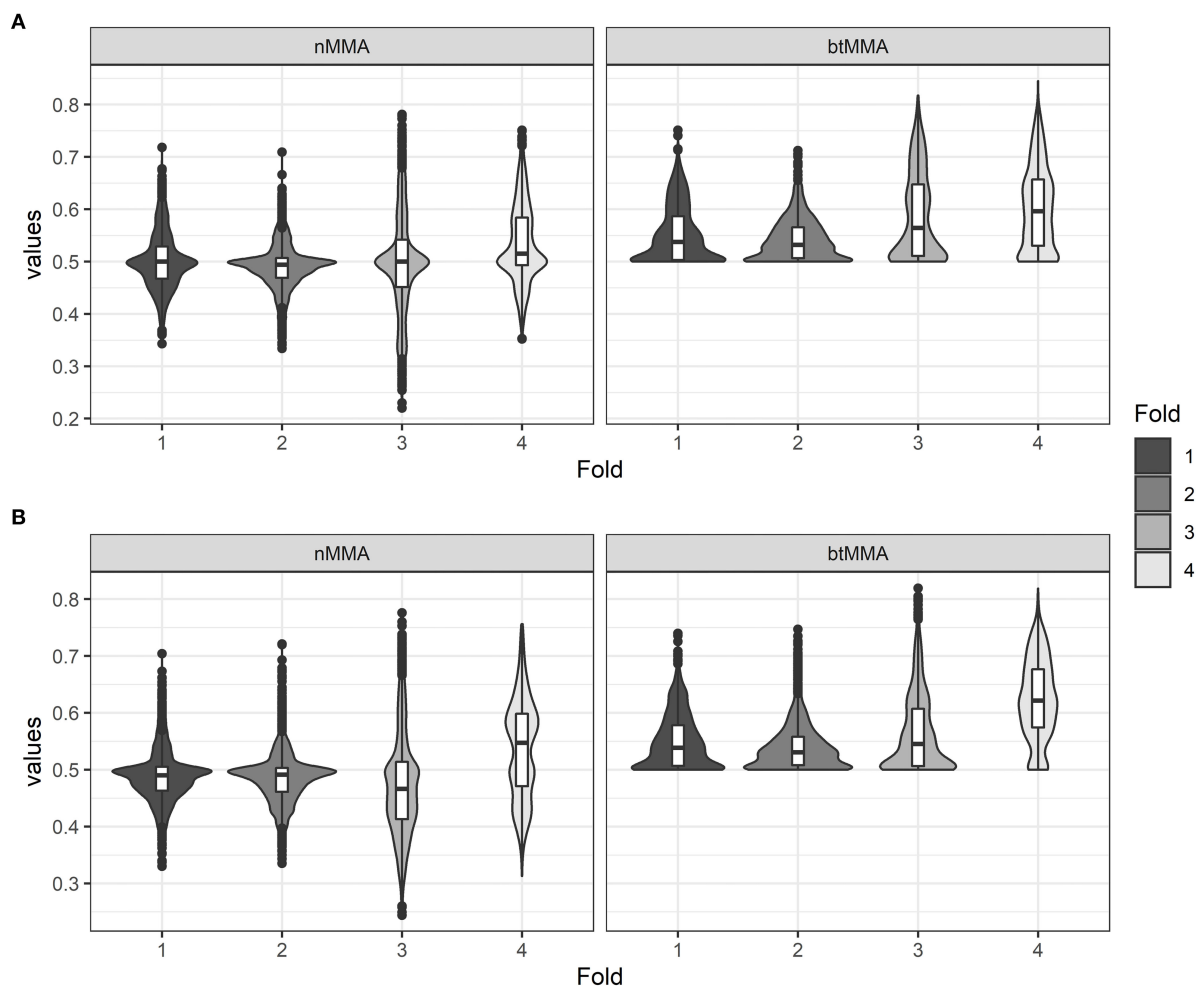
Violin plots showing the values for the varying performance metrics (nMMA: naïve major-mean accuracy, btMMA: best-threshold major-mean accuracy) for the LSTM network **(A)** and the CNN network **(B)** across the four folds of the cross-validation.

Figure 3 shows violin plots of the variation of nMMA and btMMA of the two network types across the four folds of the cross-validation. The highest average values for the performance metrics of both network types were achieved during fold 4 of the cross-validation. The summary statistics for the three metrics across CV fold and across model types are similar, and the same variation within the folds of the CV fold exists between LSTM and CNN models. Generally, the best models were achieved during fold 3 and 4 of the CV for both networks (see Table 4). In two folds of the CV, the best LSTM performed slightly better than the best CNN, while the best CNN performed slightly better than the best LSTM in the two other folds.

**Table 4:** Maximum values and parameter setting for models during CV, average over all models and for the best-fitting model of each CV fold.

**Metrics**	**Cross-validation fold**	**LSTM**						**CNN**					
		**Max**.	**Sequence length**	***N_PC_***	**Layer**	**Nodes**	**Dropout**	**Max**	**Sequence length**	***N_PC_***	**Layer**	**Filter**	**Dropout**
nMMA	1	0.718	30	512	2	Mean (N_PC_, 2)/2	0	0.704	20	128	3	16	0.2
	2	0.709	10	64	3	Sum (N_PC_, 2)	0	0.721	30	8	2	8	0
	3	0.781	30	128	2	Sum (N_PC_, 2)	0	0.776	20	64	3	8	0
	4	0.751	10	256	3	Sum (N_PC_, 2)	0.2	0.756	10	128	2	8	0
btMMA	1	0.751	20	128	2	Sum (N_PC_, 2)	0	0.740	20	128	3	16	0.2
	2	0.712	20	256	2	Mean (N_PC_, 2)/2	0.2	0.747	30	16	3	16	0
	3	0.817	30	256	1	Mean (N_PC_, 2)/2	0	0.819	30	256	3	64	0
	4	0.845	30	256	3	Sum (N_PC_, 2)	0	0.817	30	32	3	16	0.2

For the LSTM model, the maximum values achieved for the nMMA and btMMA are 78.1 and 84.5%, respectively. For the CNN model, the maximum values achieved for the nMMA and btMMA are 77.6 and 81.9%, respectively. With overall median and mean values ranging around 0.5–0.65 for the performance metrics for both model types, the majority of the tested LSTM and CNN models during cross validation did not achieve acceptable performance. However, overall max values of 0.80–0.85 indicate that useful optimal parameter settings exist for both the LSTM and CNN network. Generally, the best models were achieved with a sequence length of 30 frames, 64, 128 or 256 extracted PC's, 2 or 3 network layers and a dropout rate of 0.

While the best btMMA were consistently higher than the best nMMA for all fold of the cross-validation, the difference between these best performances (4.2 percentage points for both model types) could not be shown to be statistically significant (*p-*values: 0.29 and 0.18 for LSTM and CNN, respectively).

When analyzing the output of the cross-validation of the LSTM network using LMEM, only the *N*_*PC*_ showed a significant effect for all performance metrics and model types (see Table 5). For the LSTM network, the number of nodes used in the LSTM layer had a significant effect on both nMMA and the btMMA, while the number of layers and the sequence length only affected btMMA. For the CNN network, the filter size significantly affected btMMA, but not nMMA. Similar to the LSTM network, the sequence length affected the btMMA, while the number of layers in the CNN network affected both the nMMA and the btMMA. The implemented dropout sizes did not affect any of the performance metrics for any of the model types.

**Table 5:** Results of ANOVA analysis on the effect of the various parameter regarding data pre-processing and model architecture for nMMA and btMMA.

**Metric**	**Parameter**	**Variable**	**LSTM**					**CNN**				
			**Sum of squares**	**Mean sum of squares**	**DF**	**F-value**	***p*-value**	**Sum of squares**	**Mean sum of squares**	**DF**	**F-value**	***p*-value**
nMMA	Pre-processing	Sequence length	0.014	0.007	2	1.511	0.221	0.006	0.003	2	0.557	0.573
		N_PC_	1.171	0.146	8	30.920	< 0.001	5.237	0.748	7	160.185	< 0.001
	Model architecture	Layers	0.004	0.002	2	0.426	0.653	0.070	0.035	2	6.483	0.002
		Nodes	0.042	0.021	2	4.418	0.012	-	-	-	-	-
		1^st^ Filter-size	-	-	-	-	-	0.006	0.002	3	0.353	0.787
		Dropout	0.002	0.002	1	0.406	0.524	0.001	0.001	1	0.264	0.607
btMMA	Pre-processing	Sequence length	0.092	0.046	2	12.692	< 0.001	0.338	0.169	2	53.601	< 0.001
		N_PC_	3.585	0.448	8	123.930	< 0.001	2.258	0.323	7	112.202	< 0.001
	Model architecture	Layers	0.466	0.233	2	64.462	< 0.001	0.168	0.084	2	26.411	< 0.001
		Nodes	0.038	0.019	2	5.246	0.005	-	-	-	-	-
		1^st^ Filter-size	-	-	-	-	-	0.051	0.017	3	5.346	0.001
		Dropout	0.002	0.002	1	0.691	0.406	0.000	0.000	1	0.002	0.963

**Figure 4:**
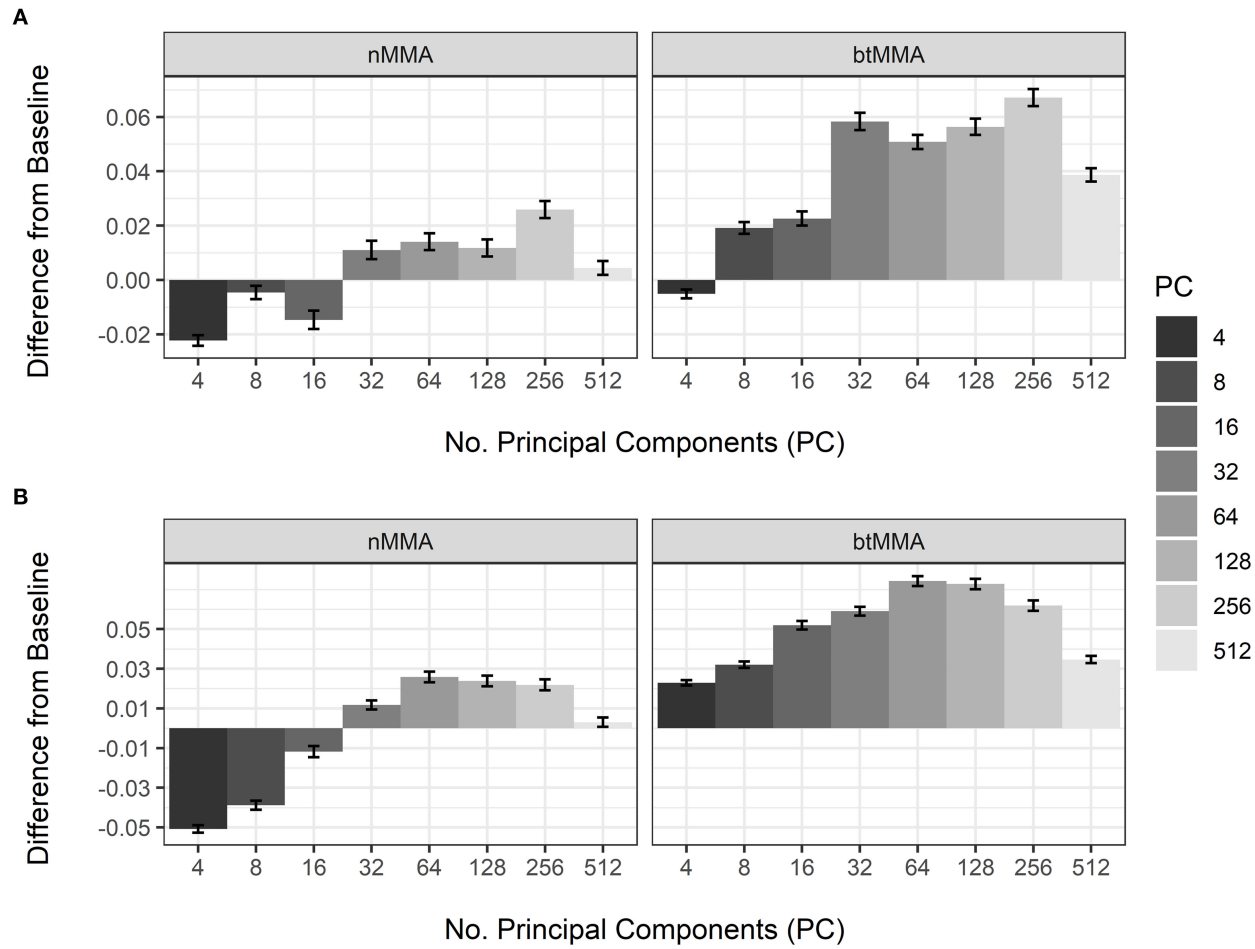
Effect of number of principal components (PC) on the variation of nMMA (naïve major-mean accuracy) and btMMA (best-threshold major-mean accuracy) of the LSTM **(A)** and the CNN **(B)** networks. The baseline is not applying principal component analyses to the extracted features.

Figure 4 shows the *post-hoc* analysis of the effect of varying *N*_*PC*_ on the performance during CV of the LSTM and CNN networks, compared to not applying PCA for dimensionality reduction (baseline). For the nMMA, using 16 or less PC was worse than not applying PCA for both the LSTM and the CNN network, while using 32 or more principal components resulted in higher nMMA values. For the btMMA, using 4 PC was worse than not applying PCA for the LSTM network. For the CNN, however, all tested values for *N*_*PC*_ resulted in higher btMMA values compared to the baseline. Table 6 shows the results of the *post-hoc* analyses of the pairwise difference of the effects of sequence length, the number of layers, the number of nodes, and the number of convolutional kernels used during the CVs of the two network types.

**Table 6:** *Post-hoc* analyses on pairwise differences of number of nodes, number of layers and sequence length on varying performance metrics during CV of the two networks.

**Variable**	**Comparison**	**LSTM**			**CNN**		
		**Metrics**	**Estimate**	***p*-value**	**Metrics**	**Estimate**	***p*-value**
Sequence length	10–20	btMMA	−0.010	0.000	btMMA	−0.010	< 0.001
	10–30		−0.008	0.001		−0.017	< 0.001
	20–30		0.002	0.838		−0.007	< 0.001
Number of layers	1–2	btMMA	0.007	0.002	btMMA	−0.011	< 0.001
	1–3		0.018	< 0.001		0.000	1.000
	2–3		0.010	< 0.001		0.010	< 0.001
	1–2		**-**		nMMA	−0.007	0.003
	1–3					−0.001	1.000
	2–3					0.006	0.013
**N**odes	Mean (N_PC_, 2) – mean (N_PC_, 2)/2	nMMA	−0.003	0.866	-		
	Mean (N_PC_, 2) – sum (N_PC_, 2)		−0.007	0.014			
	Mean (N_PC_, 2)/2 – sum (N_PC_, 2)		−0.004	0.228			
	Mean (N_PC_, 2) – mean (N_PC_, 2)/2	btMMA	0.004	0.344			
	Mean (N_PC_, 2) – sum (N_PC_, 2)		−0.003	0.483			
	Mean (N_PC_, 2)/2–sum (N_PC_, 2)		−0.007	0.009			
Filter	8–16		-		btMMA	−0.001	1.000
	8–32					0.001	1.000
	8–64					0.006	0.011
	16–32					0.003	1.000
	16–64					0.007	0.001
	32–64					0.005	0.097

Implementing networks utilizing data sequences of 20 or 30 frames compared to 10 frames significantly increased the btMMA during CV for both the LSTM networks, while for CNN network a sequence length of 30 frames compared to 10 or 20 frames increased btMMA significantly.

LSTM networks implemented with 1 layer compared to 2 and 3, and 2 compared to 3 layers, resulted in significant higher values for the btMMA. Conversely, CNN networks with 2 compared to 1 or 3 layers significantly increased nMMA and btMMA. For the number of hidden nodes, using the sum of the number of input nodes and the number of output nodes resulted in a significantly higher nMMA compared to using the mean value, but no other significant pairwise effects were found. Similarly, using the sum increased btMMA compared to using the mean divided by 2, with no other pairwise interactions present.

When testing varying number of convolutional kernels (Filters) of the CNN networks, 8 kernels in the first convolutional layer significantly increased btMMA compared all other options, except for 16, while 16 kernels were significantly better than 32 and 64, and 32 kernels was better than 64.

From the overview in Table 4 and the results of the LMEM analyses, we decided to implement final LSTM network with the following parameter:

- Sequence length: 20 and 30.
- *N_PC_*: 128 and 256.
- Number of LSTM layers: 1.
- Number of LSTM nodes: sum (*N_PC_*, 2).
- Dropout rate: of 0 and 0.2.

Similarly, the following parameters were used for the final CNN network:

- Sequence length: 10, 20 and 30.
- *N_PC_*: 128 and 256.
- Number of convolutional layers: 2.
- Number of convolutional kernels in the first convolutional layer: 8 and 16.
- Dropout rate of 0 and 0.2.

The final networks were trained on the outer training set and evaluated using the outer test set.

### 3.1. Final model evaluation

**Figure 5:**
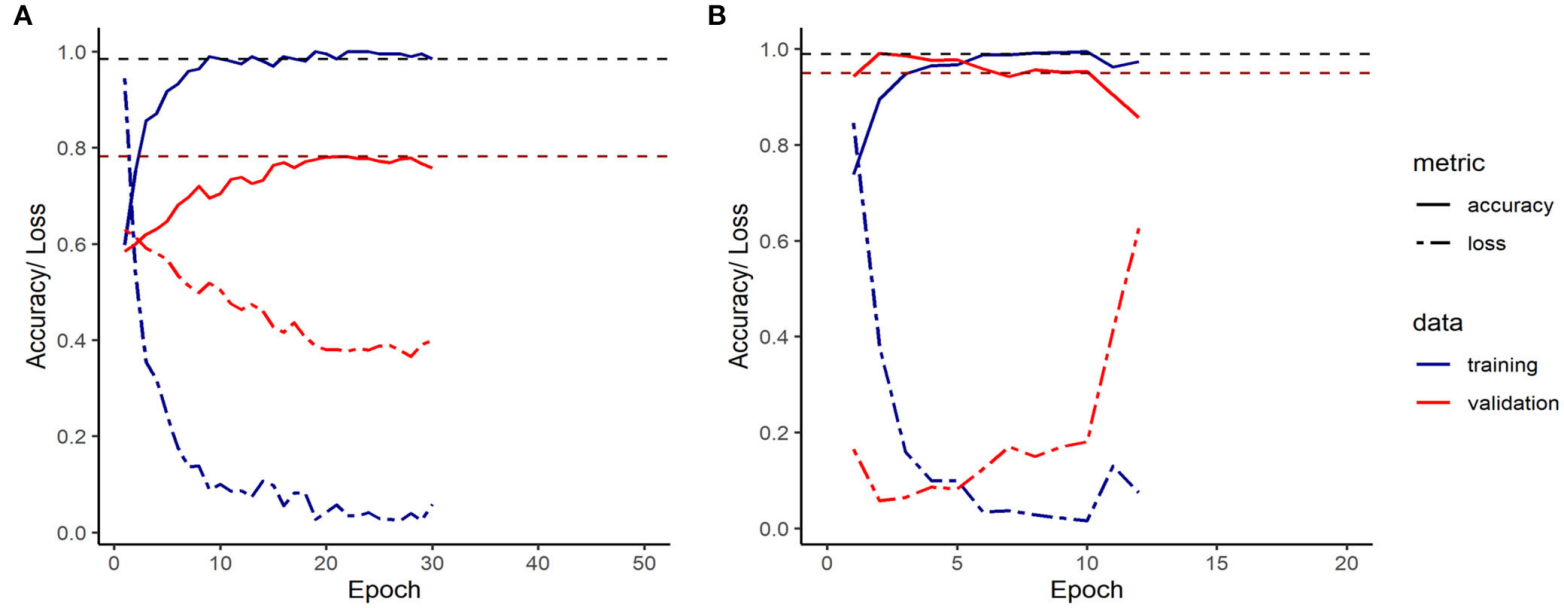
Accuracy/ Loss plot for the final LSTM **(A)** and CNN **(B)** model. (The loss function utilized was the categorical cross entropy).

Figure 5 shows the loss and accuracy on the training and validation sets during training of the final LSTM and CNN models.

On 2,110 sequences with of which 74 showed tail-biting events, the final LSTM model converged with a training's accuracy of 98.5% and a validation accuracy of 78.2% after 30 epochs, before it over-fitted. The final CNN model converged on 6,332 sequences of which 224 showed incidences of tail biting to a training's accuracy of 99.5 % and a validation accuracy of 99.0 %, before it over-fitted the data after 10 epochs.

**Figure 6:**
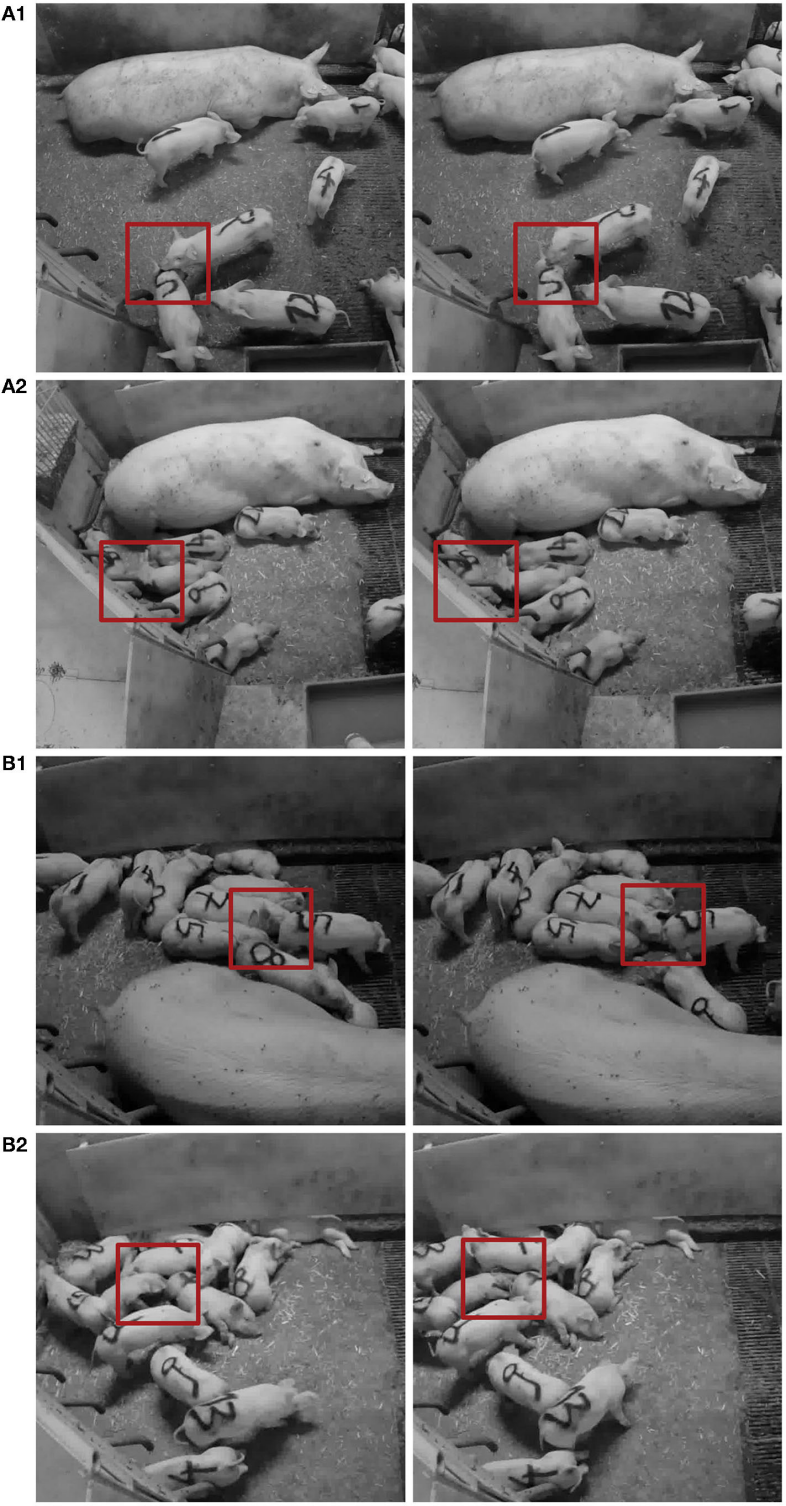
Examples of FP (1) and FN (2) classifications of tail-biting behavior of the final LSTM **(A)** and CNN **(B)** model from subsequent frames. Red rectangles indicate the area of potential misclassification.

Table 7 shows the performance metrics and the optimal parameter settings of the final LSTM and CNN network when applied to the outer test set. The ROC curve for the final LSTM model resulted in an AUC of 74%, with the best threshold for optimal class seperation at 56%. For the CNN model, the best threshold was at 12%, and the ROC curve resultet in an AUC of 67%. Figure 6 shows image examples of false positive and false negative classifications obtained when testing the final LSTM and CNN model.

**Table 7:** Performance metrics for the final LSTM and CNN model.

	**nMMA**	**btMMA**	**TP**	**FP**	**TN**	**FN**	**Seq. length**	**N_PC_**	**Nodes**	**1^st^ Filter-size**	**Layers**	**Dropout**
LSTM	71.3%	72.1%	66	956	1,080	8	30	64	Sum	-	1	0.2
CNN	64.7%	66.7%	83	141	5,624	484	10	256	-	8	2	0.2

Table 8 shows the distribution of behaviors other than tail biting (i.e., negative social behavior, nursing behavior or none of these behaviors) for the varying datasets utilized during the final model training and testing. Although there is variation in the distribution of behavioral categories, none of them is under or over represented in the utilized datasets.

**Table 8:** Descriptive statistic of the number of events of the varying behavioral categories of the dataset utilized during final model training and evaluation of the LSTM and CNN network.

**Dataset**	**LSTM**					**CNN**				
	**Sequences**	**Tail biting**	**Negative social**	**Nursing**	**No event**	**Sequences**	**Tail biting**	**Negative social**	**Nursing**	**No event**
Test 1	1,011	30	28	116	837	3,033	90	85	347	2,511
Test 2	1,099	44	87	120	848	3,299	134	256	361	2,548
Val	1,099	7	255	96	741	3,299	19	768	288	2,224
Train 1	28	13	0	7	8	91	42	0	21	28
Train 2	26	14	2	3	7	96	45	1	10	40
Train 3	36	18	0	0	18	90	50	2	0	38
Train 4	32	19	0	10	3	98	55	5	29	9
Train 5	13	2	2	2	7	30	5	1	2	22
Train 6	37	22	0	3	12	109	68	1	8	32
Train 7	22	9	0	2	11	66	25	3	8	30

The results of the prediction of the LSTM and CNN model in relation to the different behavioral categories is shown in Table 9. While the LSTM model achieves a sensitivity of 89% in predicting tail-biting events, the specificity to exclude other behaviors is lower. In contrast, the final CNN predicts tail-biting events with a sensitivity of 37%, while the specificity to exclude other behaviors reaches values above 90%.

**Table 9:** Predicted behavioral categories and corresponding specificity of the test sets for the LSTM and CNN network.

**Test set**	**Behavior category**	**LSTM**				**CNN**			
		**Predicted as no biting event**	**Predicted as biting event**	**Specificity [%]**	**Sensitivity [%]**	**Predicted as no biting event**	**Predicted as biting event**	**Specificity [%]**	**Sensitivity [%]**
Combined	Tail biting	8	66		89.19	141	83		37.05
	No tail-biting	1,080	956	53.05		5,624	484	92.10	
	No event	852	833	50.56		4,594	465	90.81	
	Negative social	74	41	64.35		329	12	96.48	
	Nursing	154	82	65.25		701	7	99.01	
Test 1	Tail biting	2	28		93.33	26	64		71.11
	No tail-biting	411	570	41.90		2,633	310	89.47	
	No event	351	486	41.94		2,213	298	88.13	
	Negative social	12	16	42.86		78	7	91.76	
	Nursing	48	68	41.38		342	5	98.56	
Test 2	Tail biting	6	38		86.36	115	19		14.18
	No tail-biting	669	386	63.41		2,991	174	94.50	
	No event	501	347	59.08		2,381	167	93.45	
	Negative social	62	25	71.26		251	5	98.05	
	Nursing	106	14	88.33		359	2	99.45	

## 4. Discussion

This study compared the achieved performance of two video-based machine learning approaches to detect tail-biting behavior in groups of pigs from sequences of video data. Previous recent research used the pigs' tail position as a proxy for tail-biting behavior (25), or deployed a tracking algorithm to detect and crop areas of pigs in close proximity to each other to be used in a CNN + LSTM network (33). Contrary to the mentioned previous research, the current study investigated the effectiveness of two deep learning methods to monitor tail-biting behavior, by analyzing sequences of image from the whole pen as a method to capture relevant temporal information in the data. We implemented a pre-trained CNN, combined with either a LSTM network (CNN-LSTM), or a CNN network (CNN-CNN) without the use of prior tracking of animal interactions. This enables the use of video data of groups of pigs for the detection of biting events, instead of data on individually located interactions. Moreover, the computational complexity as well as the workload of our methods compared to the methods reported by Liu et al. (33) are assumingly lower, making our methods more easily implementable in the field. To assess whether the methods can be used to detect tail-biting behavior in groups of pigs, and to detect the optimal framework for such methods, varying strategies regarding data pre-processing and model architecture were systematically assessed for the two methods. During cross-validation, both network types achieved similar performance results, which were obtained with similar parameter settings. Especially, the number of frames and the number of principal components, which together comprised the size of the input data, and the number of layers in the architecture were found to be relevant for the performance of both type of networks. Principal component analysis was used to reduce the dimensionality of the input vector (and thus, the computational workload), and applying PCA to the feature vectors was found to result in better performances than not applying PCA and using the raw features. This indicates that relevant spatial features exist in the data and that only specific features are necessary in the detection of tail-biting events.

Based on the respective optimal parameter settings, our final CNN-LSTM network converged with a training's accuracy of 98.5% and a validation accuracy of 78.2%, while the final CNN-CNN network converged to a training's accuracy of 99.5% and a validation accuracy of 99%. The results indicate that both methods are able to learn from the given data and that combining the pre-trained model VGG-16 with a LSTM or a secondary CNN without the use of prior tracking can be used to detect tail-biting behavior in groups of pigs. Our results are in line with findings by Liu et al. (33), who reported a validation accuracy of 92.24 % and a trainings accuracy of 98% after 20 epochs of their CNN + LSTM model utilizing the pre-trained VGG-16 model and prior tracking of animal interactions. In contrast to Liu et al. (33), the current study however utilized independent validation sets during the model optimization process and therefore the achieved validation accuracies of the methods are more robust in terms of generalizability to new data.

The current study additionally assessed the generalizability of the proposed methods in varying settings by evaluating the final models on an independent test set, which the model was not trained or validated on. The final models achieved major-mean accuracies of 71.3 and 64.7% on the final test set for the CNN-LSTM and the CNN-CNN network, respectively. This indicates that both developed models can generalize to unseen data, however, the generalization of the CNN-LSTM model appears to be superior to that of the CNN-CNN model. While the optimal number of PC for the LSTM network was 64, the CNN network performed best when utilizing 256 extracted PCs. Similarly, the optimal sequence length was 30 s for the LSTM network but only 10 s for the CNN network. As LSTM networks are capable of learning long-term dependencies between varying time steps in the data, longer sequence lengths might capture better the varying durations of tail-biting events. This might also be relevant, as situations where one pigs head is close to another pig's tail i.e., when they pass by or rest close to each other, are similar to the duration of biting events. The CNN on the contrary utilized image representations of the given sequences, which might have clouded potential long-term dependencies within the data. As the CNN uses convolutional layers to extract information of the sequence images, it may be that the current network architecture was not deep enough to grasp the information in the data.

Contrary to the study by Liu et al. (33), the current study utilized data which included other incidences of agonistic interactions (such as fighting, mounting or other biting) representative for pig production systems, adding to the generalizability of the developed models. Moreover, to capture pre-stages of tail biting which could be meaningful regarding the early detection of the behavior in pigs, a broader definition of tail biting was adapted in the current study, including milder forms of the behavior and events where the victim pig did not necessarily exhibit a reaction. As a result, most FN classifications occurred in situations, where pigs were generally in close proximity to each other, e.g., when they were lying and huddling or engaging in play/fight behavior. Similarly, FP classifications were mainly caused by one pigs head being near to another pigs tail which is similar to results by Liu et al. (33). These sitations appeared e.g., in social situations, when one pig investigated the body of another pig (see Figure 6A1), or when one pig investigated the floor with another pigs backend close (see Figure 6B1).

Furthermore, when evaluating on test data from only one pen compared to using both test pens, the sensitivity of the CNN-CNN but not of the CNN-LSTM increased from 37.1 to 71.1%. A less dramatic difference in sensitivity was also seen for the two pens when applying the CNN-LSTM approach, suggesting that something specific about Test pen 2 might be significantly outside the distribution of the other pens. The two test sets do not vary substantially in the number of other behavioral events, and the specificity for the three remaining behavioral categories is similar when using the individual test sets and the combined test set. However, in test pen 2 the sow is standing notably more and for a longer duration compared to the sows in the other videos. While it seems that the CNN-LSTM was able to handle this difference in the data, the CNN-CNN method was not able to generalize adequately when tested on test pen 2. However, as the trainings and validation accuracies for the CNN-CNN model were high, implementing the model using a larger data set might improve its generalizability to new data.

With a sensitivity of around 90% the CNN-LSTM method will detect the majority of the tail-biting events, here both including actual tail bititng and tail-in-mouth behavior, which has a huge potential in aiding a farmer in the early detection of tail biting. Being able to detect early stages of tail biting might enable the farmer to implement intervention strategies, thus preventing severe tail-biting outbreaks. This might especially be relevant, as minor tail injuries early in the production often continue to persist throughout rearing (16), and have been shown to be a significant risk factor for severe tail damage in growing/fattening pigs (39). Moreover, other visual indicators, such as hanging/tucked tails, often occur when tail biting already is prevalent in the pen or an underlying tail-biting outbreak might emerge (23, 24).

However, the current CNN-LSTM produces a substantial number of TN, i.e., in an on-farm situation will indicate that a biting event happened although there was no real biting event. Hence, although there clearly is potential for on-farm use of the proposed methods, especially the CNN-LSTM, further improvements of the methods are needed. To improve the generalizability of the proposed methods in the future, the potential of data augmentation, as well as utilizing varying pre-trained models to extract spatial features should be explored. Our study is (to our knowledge) the first of this kind of assessing the generalizability of the proposed methods to new data to detect tail-biting events in pigs from video recordings. This is of high value, as a good generalizability entails that such models can be used with varying environmental set-ups and at different pig units. Future studies should evaluate the generalizability of the methods to different farm setting and to animals at different stages of the pig production.

## 5. Conclusion

We explored the applicability of CNN-LSTM and of CNN-CNN frameworks with our available data, and systematically assessed the performance and generalizability of the developed models regarding varying data pre-processing and model architecture parameters. The results of the study indicate that the proposed methods can detect tail-biting behavior from video sequences of entire pig pens, with the CNN-LSTM model being superior in terms of generalizing to unseen data, compared to the CNN-CNN model. The study also found that implementing principal component analyses on the extracted spatial feature vectors and using a limited number of PCs as input to the networks can increase the performance, compared to using all extracted features. Due to its lower complexity and computational workload and with a sensitivity of 89% on new data, the CNN-LSTM seems to be the most promising method considering on-farm implementation.

## Data availability statement

The data analyzed in this study is subject to the following licenses/restrictions: The data utilized in this study consists of video recordings of entire pig pens. The data has not yet been made publicly available. Requests to access these datasets should be directed to FH, fh@sund.ku.dk.

## Ethics statement

Ethical review and approval was not required for the animal study because this study utilizes only data sources, obtained by another study. Additionally, the data sources (videos) utilized in this study were obtained without interference with the animals or the farm routine, and data was collected with the permission of the farmer/farm manager.

## Author contributions

FH contributed to data collection and analyses and wrote the first draft of the manuscript. FH and DJ contributed to the development of the ML code, revising the manuscript, and reading and approving the submitted version.
